# Suprasternal Bronchogenic Cyst Masquerading as Thyroid Swelling: A Case Report

**DOI:** 10.7759/cureus.83870

**Published:** 2025-05-10

**Authors:** Aditya Sharma, Priyansh Garg, Satyendra K Tiwary, Vivek K Katiyar, Puneet Kumar

**Affiliations:** 1 Department of General Surgery, Institute of Medical Sciences, Banaras Hindu University, Varanasi, IND

**Keywords:** bronchogenic cysts, congenital disease, head and neck tumors, head neck surgery, open excision

## Abstract

Abnormal foregut budding during early embryonic development results in bronchogenic cysts, which are uncommon congenital anomalies that usually develop along the tracheobronchial tree, in the mediastinum, or inside the lung parenchyma. Bronchogenic cysts, which are lined with respiratory epithelium and filled with fluid or mucus, are frequently asymptomatic but might develop compressive symptoms or recurrent infections if they increase, get infected, or occasionally rupture. Imaging is usually used to make the diagnosis; magnetic resonance imaging or computed tomography can show a well-defined cystic lesion. Asymptomatic cysts can occasionally be treated with observation, but severe or symptomatic cysts typically need to be surgically removed because of the risks of infection, compromised breathing, and, in rare cases, malignant transformation.

This case report highlights a 30-year-old woman presenting with a longstanding suprasternal swelling initially suspected to be thyroid-related. The clinical findings, diagnostic evaluation, and surgical management are described.

## Introduction

Bronchogenic cysts are uncommon congenital lesions that arise from abnormal development of the primitive foregut, leading to the formation of cystic structures lined by respiratory epithelium [1-3]. While typically located in the mediastinum, ectopic bronchogenic cysts can occur in the cervical region, including the suprasternal area. Due to their midline location, these cysts can be mistaken for thyroid swellings, necessitating careful differential diagnosis to ensure appropriate management [2,3]. With a male-to-female ratio of 4:1 and a frequency of 1 in 42,000 to 68,000, bronchogenic cysts are rare congenital anomalies of the tracheobronchial tree [1,3]. The age of presentation is typically the younger population. In this case report, we present a case of a suprasternal bronchogenic cyst in a 30-year-old woman.

## Case presentation

**Figure 1:**
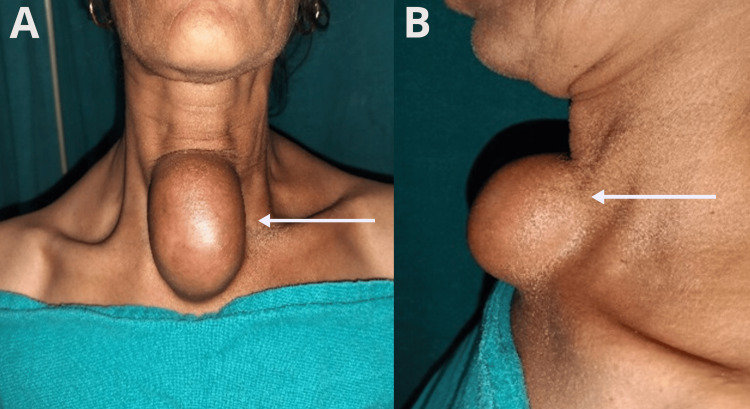
(A) Clinical image showing the exact location of the suprasternal cyst; (B) oblique view of the swelling.

A 30-year-old woman presented to the surgery outpatient department with a swelling in the suprasternal region first noticed 20 years ago. Initially asymptomatic, the swelling had gradually increased in size and was associated with mild pain for the past three months. The patient denied symptoms of dysphagia, dyspnea, or any previous medical history and surgical management. On examination, midline swelling was approximately 7×5 cm, soft, cystic, slightly tender, and mobile with well-defined borders. The overlying skin was intact without erythema or discharge. It did not move with swallowing or tongue protrusion, distinguishing it from thyroid or thyroglossal duct anomalies. There were no palpable cervical lymph nodes, and the other system examination was within normal limits, as shown in Figure 1.

**Figure 2:**
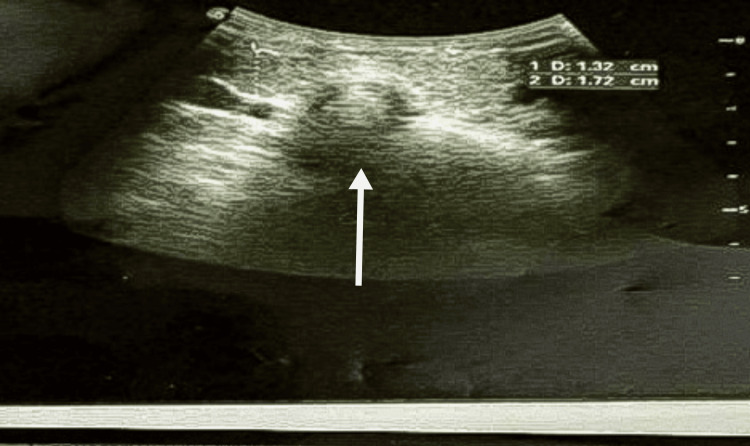
Ultrasonography of the suprasternal swelling.

The ultrasonography of the neck revealed a well-defined, hypoechoic, heterogeneous cystic lesion measuring approximately 6×4×6 cm, located superficial to the sternum and separate from the thyroid gland. No internal septations, calcifications, or vascularity were observed. The thyroid gland was visualized as normal, with no pathological changes, as shown in Figure 2.

**Figure 3:**
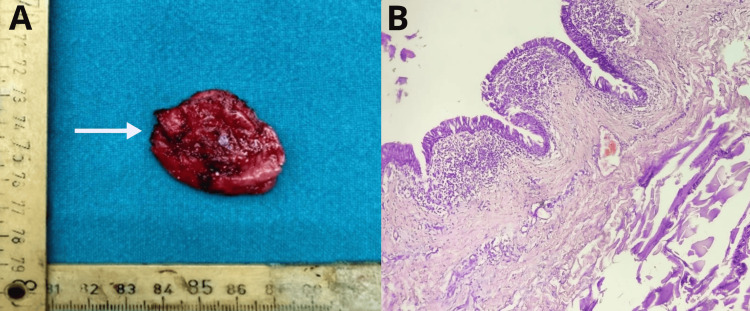
(A) Resected specimen of the bronchogenic cyst; (B) histopathological findings.

The fine-needle aspiration cytology (FNAC) was also performed and yielded thick, whitish material. Cytological analysis revealed anucleate squamous cells and keratinous debris, findings consistent with an epidermal inclusion cyst. There were no malignant cells or atypia noted in FNAC. Under general anesthesia, the patient underwent the surgical excision of swelling. Over the swelling, a collar incision was made transversely along the skin crease. A well-encapsulated cystic mass attached to the subcutaneous tissues but distinct from deeper structures, such as the thyroid gland, was noted after careful dissection. Hyperextension of the neck was done, and retraction of the cyst upwards allowed access to the lower edge. The patient was discharged on the second postoperative day after an uneventful postoperative period. The histopathological report showed a cystic tissue piece lined by ciliated columnar to squamous epithelium. The underlying fibroconnective tissue shows moderate to dense inflammatory infiltrate with mild lymphoid aggregate. There was no evidence of malignancy in the sections examined, confirming the diagnosis of a bronchogenic cyst. The patient was followed initially on a weekly basis and thereafter monthly for the next four months. She did well in her follow-up period. The resected specimen of the bronchogenic cyst along with the histopathological findings is shown in Figure 3.

## Discussion

The literature shows bronchogenic cysts are more prevalent in men and typically manifest with symptoms in infancy [2,4,5]. While they are most commonly located in the mediastinum, ectopic bronchogenic cysts can occur in unusual locations, including the suprasternal region. The location of these is usually presternal or at the suprasternal notch of the neck region. They may be misdiagnosed with thyroid swellings or other midline neck masses because of their midline placement [5,6].

Branchial cleft cysts, epidermal inclusion cysts, thymic cysts, thyroid cysts, dermoids and lymphangiomas, cystic teratomas, thyroglossal duct cysts, bronchogenic cysts, and cystic hygromas are among the differential diagnoses for congenital cervical cysts in children [6]. Imaging tests, FNAC, and clinical examination are crucial methods of differentiating bronchogenic cysts from these other conditions. As seen in the current example, where imaging investigations indicate the presence of a dermoid cyst, imaging is crucial, but histological examination is necessary to make the definitive diagnosis [7].

Complete removal is important to avoid recurrence and is the treatment of choice for bronchogenic cysts to prevent potential complications, including infection, rupture, or malignant transformation. Following surgery, histopathological confirmation is necessary for a conclusive diagnosis. A fibrous connective tissue wall with cartilaginous plates and seromucous subcutaneous glands is covered by respiratory epithelium, more precisely ciliated pseudostratified cylindrical epithelium, which lines these cysts [7,8].

## Conclusions

The important aspect of taking bronchogenic cysts into account while making a differential diagnosis for midline neck swellings is demonstrated by the present case. Accurate diagnosis based on clinical examination, ultrasound, and FNAC is crucial to guide appropriate surgical management and avoid misdiagnosis as thyroid pathology.

## References

[1] Aktoğu S, Yuncu G, Halilçolar H, Ermete S, Buduneli T (1996). Bronchogenic cysts: clinicopathological presentation and treatment. Eur Respir J.

[2] McAdams HP, Kirejczyk WM, Rosado-de-Christenson ML, Matsumoto S (2000). Bronchogenic cyst: imaging features with clinical and histopathologic correlation. Radiology.

[3] St-Georges R, Deslauriers J, Duranceau A (1991). Clinical spectrum of bronchogenic cysts of the mediastinum and lung in the adult. Ann Thorac Surg.

[4] Ortiz RJ, Reusmann A, Boglione MM (2023). Bronchogenic cyst: lessons learned in 20 years of experience at a tertiary pediatric center. J Pediatr Surg.

[5] Lardinois D, Gugger M, Ris HB (1999). Bronchogenic cyst of the left lower lobe associated with severe hemoptysis. Eur J Cardiothorac Surg.

[6] Tu C, Zhu J, Shao C (2016). Gastric bronchogenic cysts: a case report and literature review. Exp Ther Med.

[7] Cohn JE, Rethy K, Prasad R, Mae Pascasio J, Annunzio K, Zwillenberg S (2020). Pediatric bronchogenic cysts: a case series of six patients highlighting diagnosis and management. J Invest Surg.

[8] Durhan G, Ardali Duzgun S, Akpınar MG, Demirkazık F, Arıyürek OM (2021). Imaging of congenital lung diseases presenting in the adulthood: a pictorial review. Insights Imaging.

